# A macrophage-T cell coculture model for severe tissue injury-induced T cell death

**DOI:** 10.1016/j.xpro.2021.100983

**Published:** 2021-12-01

**Authors:** Jie Zhu, Jiayu Cao, Arthur Liesz, Stefan Roth

**Affiliations:** 1 Institute for Stroke and Dementia Research (ISD), University Hospital, LMU Munich, Munich, Germany; 2 Munich Cluster for Systems Neurology (SyNergy), Munich, Germany

**Keywords:** Cell Biology, Cell culture, Cell isolation, Health Sciences, Immunology

## Abstract

A key observation of tissue injury, such as stroke and burn, is a state of systemic immunosuppression characterized by loss of T cells and rise of infections. Here, we present an *in vitro* model for cell-cell interactions between innate (macrophages) and adaptive (T cells) immune cells. This protocol facilitates bone marrow-derived macrophages (BMDMs) and splenic T cells in a coculture model. The procedure mimics injury-induced T cell death, which is driven by inflammasome activation in macrophages.

For complete details on the use and execution of this protocol, please refer to Roth et al. (2021).

## Highlights

• Coculture model of murine bone marrow-derived macrophages and splenic T cells
• Modeling immune cell-cell interactions between innate and lymphoid immune cells
• Analysis of cell death receptor-ligand interactions between macrophages and T cells
• Serum from mice with severe tissue injury as initial stimulus for the coculture

## Graphical abstract

**Figure:**
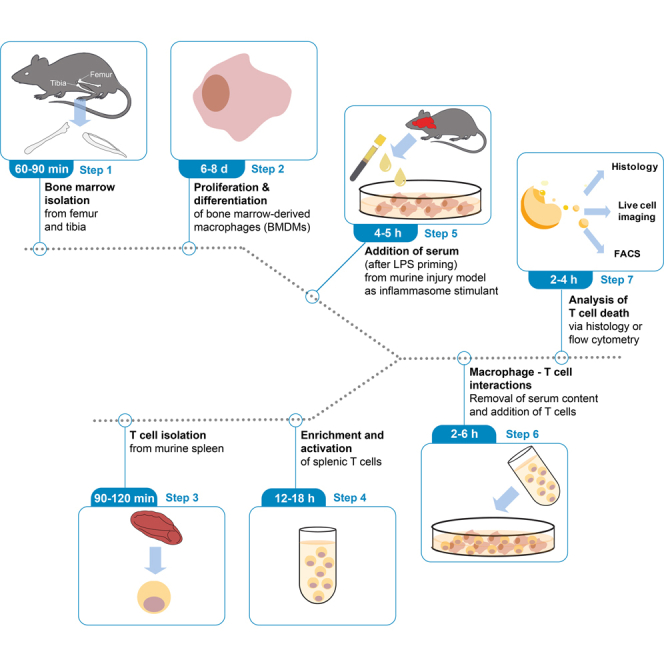

## Before you begin

### Preparation of consumables, surgery tools and kits

**Timing: 20 min (autoclave: 90 min)**

1. 96-well plate preparation for T cell culture.
  a. 96-well U-bottom plates are coated with anti-CD3e antibodies overnight at 4°C (15–18 h; please see also Wang and Ai, 2021). The stock antibody (0.5 mg/mL) is diluted 1:100 in sterile PBS and 30 μL of CD3e solution is pipetted in each well.
2. Surgical tools for organ removal.
  a. For removal of femurs, tibiae and spleen properly clean (or ideally autoclave) a pair of extra fine scissors and 2 pair of forceps.
3. Preparation of consumables for BMDM culture
  a. 15 cm culture dishes are needed for the differentiation of BMDMs.
  b. 96-well flat-bottom plates (see key resources table) are needed for seeding differentiated BMDMs for inflammasome stimulation and cell–cell interaction.

### Preparation of cell culture media and solutions

**Timing: 30 min**

All recipes can be found in “materials and equipment”.

4. Bone marrow-derived macrophage medium
  a. Mix DMEM (+GLutaMAX) with 10 % inactivated (56°C for 30 min) fetal bovine serum (FBS) and 1 % Gentamycin (stock: 50 mg/mL).
  b. Before usage heat up to 37°C in water bath.
5. Inflammasome stimulation medium
  a. For stimulation of the BMDMs’ inflammasome, collect murine serum from mice which received an experimental stroke (Figure 1) and add it to BMDM medium. The final solution contains 25 % murine stroke serum.

**Figure 1:**
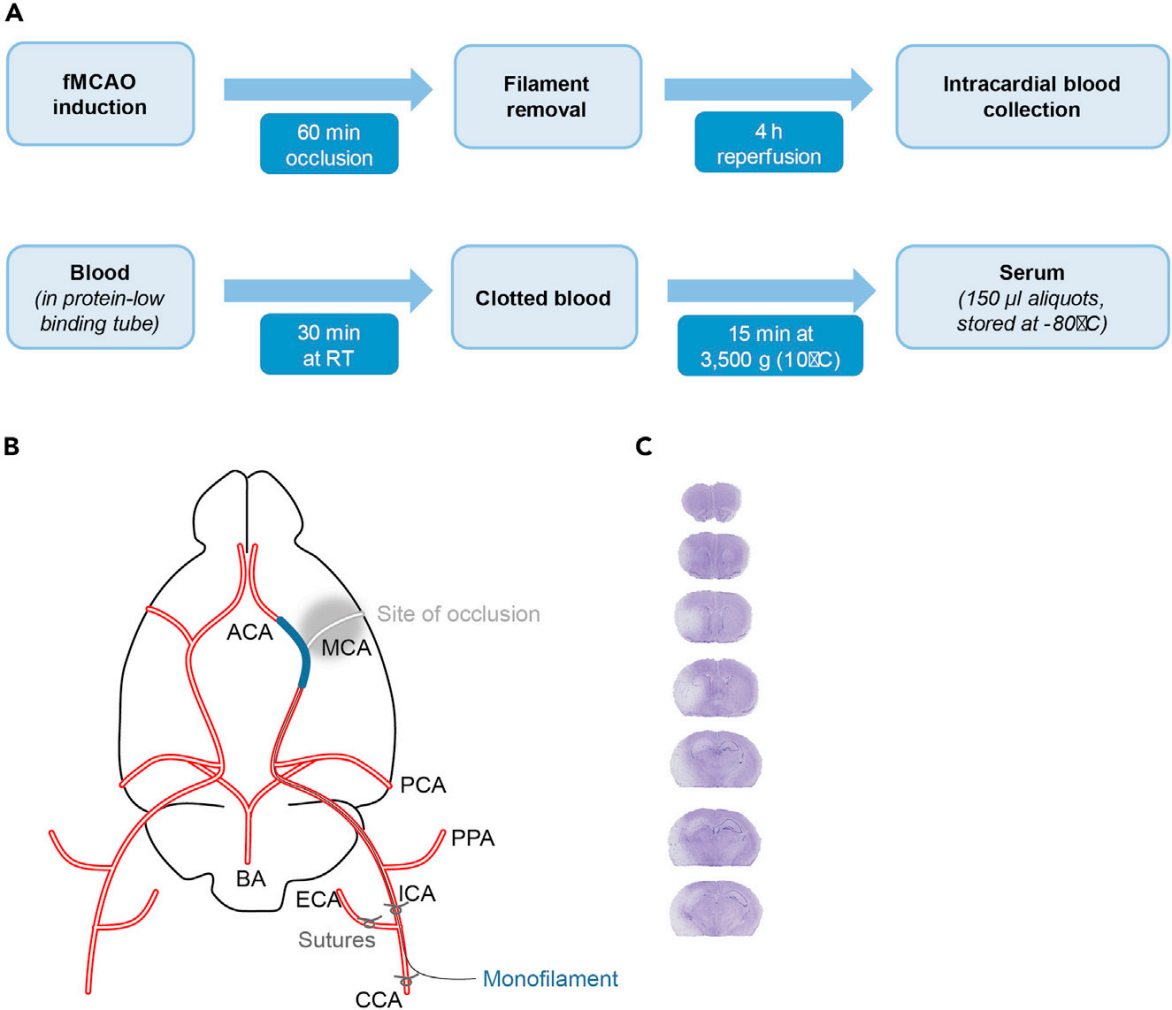
Serum collection from mice with acute tissue injury (A) In our model mice received an experimental stroke (filament-induced occlusion of the middle cerebral artery (fMCAO; 60 min of ischemia)) and were then sacrificed 4 h after reperfusion (5 h after the start of the surgery). Mice were sacrificed and blood was drawn intracardially with a 24 gauge needle. Blood was stored in 1.5 mL protein-low binding tubes and kept for 30 min at room temperature (RT) for clotting. Tubes were then centrifuged at 3,500 *g* for 15 min. Serum (=supernatant) was then collected, aliquoted and immediately stored at −80°C until further usage. (B) Schematic of the circle of willis: For the model utilized in the manuscript, a silcon-coated mono filament was inserted in the common carotid artery (CCA) and pushed up (via the internal carotid artery (ICA)) to occlude the middle cerebral artery (MCA). (C) Representative cresyl violet staining of coronal brain sections. The healthy tissue is stained in violet, the lesion areas remain white. Abbreviations: ACA = anterior cerebral artery; BA = basiliar artery, CCA = common carotid artery, ECA = external carotid artery, PCA = posterior cerebral artery, PPA = Pterygopalatine artery.
6. T cell medium
  a. Mix RPMI 1640 complete with 10% fetal bovine serum (FBS) and 1 % Penicillin/Streptomycin (Stock: 10,000 U/10,000 U)
  b. Before usage add β-mercaptoethanol to a final concentration of 5 nM and heat the medium up to 37°C.

### Preparation of mice

All animal experiments were performed in accordance with the guidelines for the use of experimental animals and were approved by the respective governmental committee (Regierungspraesidium Oberbayern).

C57BL6/J Mice were used for bone marrow cell and serum collection after experimental stroke. The mice had an age of 6–12 weeks, and both, female and male were used.

***Optional:*** GFP+ T cells can be used in the below described coculture procedure. For this approach B6 Actin-eGFP (C57BL/6-Tg(CAG-EGFP)1Osb/J; JAX stock: 003291) mice were used. The mice had an age of at least 8 weeks and both, female and male, mice were used.

## Key resources table

**Table:** 

REAGENT or RESOURCE	SOURCE	IDENTIFIER
**Antibodies**		

Anti-mouse CD3e (FITC/APC; 17A2, working dilution for FACS: 1:250 )	Invitrogen	11-0032-82/17-0032-82
Anti-mouse CD45 (eFluor450; 30-F11, working dilution for FACS: 1:250)	Invitrogen	18-0451-82
Anti-mouse CD11b (PerCP-Cy5.5/PE-Cy7; M1/70; working dilution for FACS: 1:250)	Invitrogen	45-0112-82/25-0112-82
Anti-mouse CD4 (PerCP-Cy5.5; RM4-5; working dilution for FACS: 1:250)	Invitrogen	45-0042-82
Anti-mouse F4/80 (FITC/APC eF780; BM8; working dilution for FACS: 1:250)	Invitrogen	11-4801-82 / 47-4801-82
Anti-mouse MHCII (PE; NIMR4; working dilution for FACS: 1:250)	Invitrogen	12-5322-81
Anti-mouse CD178 (APC; MFL3; working dilution for FACS: 1:250)	Invitrogen	17-5911-82
Anti-mouse CD16/32 (Fc Blocker; 93; working dilution for FACS: 1:1000)	Invitrogen	14-0161-82
Anti-mouse CD28 Monoclonal Antibody (funct. grade / purified; working concentration 0.1 μg/μL)	Invitrogen	16-0281-82

**Chemicals, peptides, and recombinant proteins**		

DMEM+GlutaMAX(4,5 g/I D-Gluc./ Pyruvate)	GIBCO	31966-021
Gentamicin (50 mg/mL)	GIBCO	15750-045
RPMI1640 (L-Glutamine / 25mM HEPES)	GIBCO	52400-025
Penicilin / Streptomycin	GIBCO	15140-122
Fetal calf serum (FCS)	GIBCO	105000-064
Recombinant Murine M-CSF	PeproTech	315-02
2-Mercaptoethanol	GIBCO	31350010
LPS from E.coli	Adipogen	IAX-100-013-M001
HBSS	GIBCO	14175-053
FACS staining buffer	Thermo Fisher Scientific	00-4222-26
Cresyl Violet acetate (working concentration 0.5 %)	Sigma-Aldrich	C5042-10G

**Critical commercial assays**		

T cell enrichment MagniSort	Thermo Fisher Scientific	8804-6820-74

**Experimental models: Organisms/strains**		

C57BL6/J (male or female; 6–12 weeks old)	Charles River Laboratories	000664
C57BL/6-Tg(CAG-EGFP)1Osb/J (male or female; >8 weeks old)	The Jackson Laboratory	003291

**Experimental models: Cell lines**		

NCTC clone 929 [L cell, L-929]	ATCC	CCL-1

**Software and algorithms**		

BD FACSuite	Beckton Dickinson	N/A
Microsoft Excel	Microsoft Corporation	N/A
FlowJo v.10.6	Treestar Inc.	N/A
GraphPad Prism 6	GraphPad Software, Inc.	N/A
Creative Suite 6	Adobe	N/A
ImageJ Software	NIH, US	N/A

**Other**		

40 μm cell strainer	FALCON	352340
50 mL Conical Centrifuge Tubes	FALCON	352070
4 mL Round-Bottom Polystyrene Test Tubes	FALCON	352052
Cell culture dish	Thermo Fisher Scientific	168381
Cell Scraper	FALCON	353086
Counting slides	Bio-Rad Laboratories	145-0011
Forceps	Fine Science Tools	11253-25
Scissors	Fine Science Tools	14090-09
Automated Cell counter	Bio-Rad Laboratories	TC20
Neubauer counting chamber	CLS Medizintechnik	A51005
96-Well, Cell Culture-Treated, U-Bottom Microplate	Thermo Fisher Scientific	3799
Injekt F Syringe	B. Braun	9166017
Cell culture dish	CELLSTAR	639960
5mL FACS tube	FALCON	352052

## Materials and equipment

Preparation of buffers and solutions.

### Murine stroke serum collection and storage

Mice received a transient filament-induced occlusion of the middle cerebral artery (fMCAO, e.g., Engel et al., 2011; Figures 1A and 1B). Serum from mice receiving an experimental stroke is collected after 4 h of reperfusion time. The rodents are sacrificed and blood is collected transcardially. Representative staining of the infarction with cresyl violet (0.5 %) provides an idea of the severity of the neural lesion (Figure 1C).

**Table:** Bone marrow-derived macrophage medium

Reagent	Final concentration	Amount
DMEM	N/A	445 mL
FBS	10%	50 mL
Gentamycin	1%	5 mL

Store at 4°C for a maximum of 2 months; heat up to 37°C before usage.

**Table:** Inflammasome stimulation medium

Reagent	Final concentration	Amount (per well)
DMEM	N/A	207 μL
FBS	10%	30 μL
Gentamycin	1%	3 μL
Murine stroke serum	20%	60 μL

Needs to be prepared freshly and provided at 37°C for usage.

**Table:** T cell culture medium

Reagent	Final concentration	Amount
RPMI 1640	N/A	445 mL
FBS	10%	50 mL
Penicilin/Streptomycin	1%	5 mL

Store at 4°C for a maximum of 2 months; before usage, add β-mercaptoethanol to a final concentration of 5 nM and heat up to 37°C.

## Step-by-step method details

Overview experimental schedule

### Generation of murine bone marrow-derived macrophages (BMDMs)

**Timing: Procedure: 60–90 min; Proliferation and differentiation: 6 days**

The following steps provide the procedure of collection and differentiation of bone marrow-derived cells into BMDMs.

**Figure 2:**
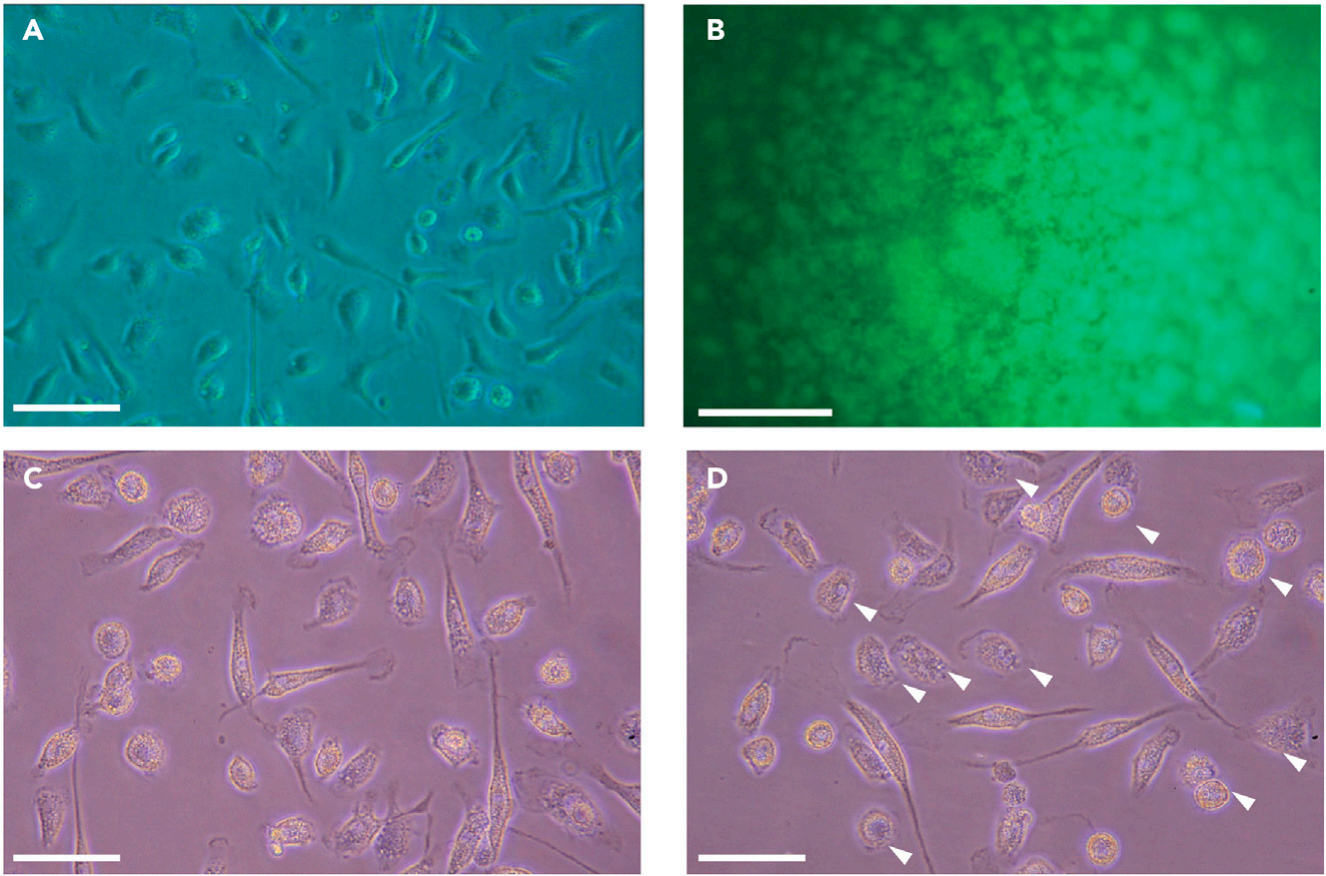
Culturing T cells and BMDMs (A) Differentiated, confluent BMDMs 6 days after culturing the initial bone marrow-dervied cells. (B) Representative image of cultured splenic T cells in an anti CD3/CD28-coated U-bottom well. (C) Differentiated BMDMs before being primed with 100 ng/mL LPS. (D) Differentiated BMDMs after the first 2 h LPS priming. Loss of ramifications and the swelling of the cell body indicates successful priming (white arrowheads, shown before in e.g., Kawakami et al., 2016). In panel (A), (C) and (D) scale bars represent 100 μm. In panel B scale bar represents 1 mm.

1. Femur and tibia collection.
  a. Euthanize BL6/J mouse, disinfect and fixate in supine position.
  b. Carefully expose femur and tibia from surrounding muscles and tissue (a detailed protocol can be found in Toda et al., (2020)).
  c. Store prepared bones in 10 mL of ice-cold PBS for transfer to laminar air flow.
2. Collection and straining of bone marrow-derived cells.
  a. Prepare 50 mL conic tube and 40 μm strainer (one per mouse).
  b. Remove the epiphyses from femurs & tibias.
  c. Flush out the bone marrow from femurs and tibiae onto the 40 μm strainer.
    i. Use a 23 G needle on a 1 mL syringe (see materials and equipment) filled with sterile PBS, position the needle in the bone shaft and carefully flush until bone marrow is mobilized. Repeat procedure until all bone marrow is on the strainer.
  d. Use syringe plunger and additional 10 mL PBS to flush the remaining cells through the strainer into the conic tube. Afterward fill up volume with PBS to 20 mL.
  e. Wash cells by centrifuging the tube at 500 *g* for 8 min at room temperature (RT; 20°C–25°C).
  f. Resuspend in 20 mL PBS and wash again (500 *g* for 8 min).
3. Seeding of bone marrow-derived cells in culture dishes for differentiation.
  a. Resuspend with 2 mL BMDM medium and use a Neubauer counting chamber or an automated cell counter (see key resources table) to acquire cell numbers.
    i. In our experience, 2 femurs and tibiae from one mouse provide approx. 6–10 × 10^7^ cells).
  b. Prepare a cell suspension with 3–5 × 10^7^ bone marrow-derived cells in 2 mL BMDM medium (1.5–2.5 × 10^7^ cells per mL) and seed them on a 15 cm cell culture dish (see key resources table).
  c. Provide 150 ng of M-CSF to each culture dish, add additional 13 mL of BMDM medium and place the cells in the incubator at 37°C. The final concentration of M-CSF is 10 ng/mL.
4. Differentiation and proliferation of BMDMs from day 2 to 6.
  a. Provide 150 ng of M-CSF and 3 mL BMDM medium to each culture dish daily for another 4 continuous days. The culture should provide healthy macrophages with a ramified, mononuclear morphology after 6–7 d (as seen in Figure 2A).

**CRITICAL:** For setting up a pure macrophage culture it is important to observe the cells daily (ramified morphology, loss of other bone marrow-derived cells over time, cell confluency (Figure 1A)). Alternatively, L929 (murine connective tissue fibroblast cell line; ATCC, US; see key resources table) -conditioned medium can be used as a substitute for recombinant M-CSF.

### Harvest and seeding of BMDMs in 96-well plates

**Timing: 30 min; day 7 after BMDM culture start**

In this step, the differentiated BMDMs will be seeded on 96-well plates 1 day prior to the coculture approach. Carefully check the ramified morphology and adhesion of BMDMs, as indicator for viability, before starting the following steps.

5. Harvest of BMDMs from the 15 cm culture dishes.
  a. PBS and BMDM medium needs to be warmed up to 37°C.
  b. Remove the medium from the culture dish and carefully wash the dish twice by slowly pouring 10 mL pre-warmed PBS on the dish, panning and removal of the PBS.
  c. Add 10 mL pre-warmed PBS and detach the cells by gentle scraping with cell scraper (see key resources table), collect the cell suspension in conic 50 mL tube. Check the dish visually for remaining cells. Avoid intensive scraping and formation of bubbles, to receive healthy cells.
    ***Optional:*** Add another 10 mL PBS to the dish, repeat scraping and collect the cell suspension in the same conic 50 mL tube.
  d. Add the cell suspension volume up to 20 mL by eventually adding PBS and wash at 300*g* for 7 min at RT.
  e. Wash again (300 *g* for 7 min, RT).

6. Seeding of BMDMs into 96-well plate.
  a. Discard supernatant and resuspend the cells in 2 mL BMDM medium.
  b. Acquire cell numbers and seed 1 × 10^5^ BMDMs per well into a flat-bottom 96-well plate (see key resources table).
  c. Place the cells in the incubator at 37°C and let them settle and ramify overnight (12–18 h).

### T cell isolation from murine spleen

**Timing: 90–120 min; Expansion: 1 day**

In this step, splenic T cells will be enriched from murine spleen. This is achieved by using a magnetic bead-based commercial T cell negative selection kit. Make sure that you have prepared anti-CD3ε-coated 96-well plates before starting isolation. Coating with anti-CD3e antibody is essential for functional T cell culture.

7. Spleen collection.
  a. Euthanize Bl6/J mouse, disinfect and fixate in supine position.
  b. Open the abdomen and expose the spleen from surrounding fat tissue.
  c. Remove the spleen and store in 10 mL of ice-cold PBS for transfer to laminar air flow.

8. Collection, straining and enrichment of splenic T cells.
  ***Note:*** The T cell isolation was done with the MagniSort T cell enrichment kit (ThermoFisher, US; see key resources table). Volumes and incubation times are according to the manufacturer’s protocol.
  The used antibody cocktail contains: Anti-mouse CD11b, CD19, CD24, CD45R, CD49b, Ly6G and TER-119 according to the manufacturer’s data sheet. Using alternative kits for T cell enrichment/isolation is possible.
  a. Prepare conic 50 mL tube and 40 μm strainer.
  b. Mince the spleen using scissors and carefully press tissue through the 40 μm strainer with a syringe plunger while continuously rinsing with a least 10 mL PBS
  c. Fill up volume to at least 20 mL volume and wash the suspension at 300 *g* for 10 min at RT.
  d. Acquire cell numbers and titrate the concentration to 10^7^ cells per 100 μL.
  e. Transfer the whole cell suspension into a 5 mL “FACS” tube (see key resources table)
  f. Add 20 μL of the T cell enrichment antibody cocktail (please see **“Note”** for the T cell enrichment kit) per 100 μL cell suspension, briefly vortex at least 3 times and incubate 10 min at RT. Red blood cell lysis is not required here, the antibody cocktail of the enrichment kit implements anti-TER-119, removing red blood cells.
  g. Bring the suspension volume up to 4 mL with Hank’s balanced salt solution (HBSS, see key resources table) and wash at 300 g for 8 min at RT.
  h. Discard supernatant and resuspend with 100 μL HBSS per 10^7^ cells.
  i. Add 20 μL of the magnetic bead-labeled antibodies per 100 μL cell suspension, briefly vortex at least 3 times and incubate for 5 min at RT.
  j. Bring the volume up to 2.5 mL with HBSS and place the FACS tube into the supplied magnet (see “**Note**”) for 5 min at RT. In this critical step separation of magnetic bead-labeled immune cells and unlabeled T cells is conducted.
  k. Decant carefully the suspension from the “magnetized” tube into a fresh 4 mL FACS tube (=enriched T cells).
    ***Optional:*** Remove the remaining tube (= removed splenocytes, antibody-labeled) and use the cells for quality control via flow cytometry.

9. Seeding T cells and initiation of proliferation.
  a. Wash the enriched T cells at 300 *g* for 8 min (RT).
  b. Acquire cell numbers and titrate to a concentration of 3 × 10^5^ cells per 200 μL with T cell medium (see “before you begin”).
  c. Place 200 μL T cell suspension per well onto a U-bottom 96-well plate (pre-coated with CD3e antibody; see “before you begin” and key resources table).
  d. After letting the cells settle for 15 min add CD28 antibody (10 μL; 0.1 μg/μL) to the T cells.
  e. Place the cells overnight (12–18 h) into an incubator at 37°C for settling.
  f. Control the viability of the T cells before starting the coculture approach (Figure 1B).

### Coculture procedure

**Timing: 9–10 h**

**Timing: 10 min; 4 h of priming for step 10**

**Timing: 30 min for step 11**

**Timing: 10 min; 15–30 min stimulation for step 12**

**Timing: 10 min; 2–6 h incubation, variable incubation times possible for step 13**

Here we describe the procedure of coculturing BMDMs, primed with LPS and stimulated with murine stroke serum, together with (anti-CD3 + anti-CD28 stimulated) splenic CD3+ T cells. These are the most critical steps, which should be well prepared (pre-warmed medium etc.).

Critical is to control adhesion and ramified morphology of the macrophages. Also the T cells need visual control: Dark spots in the T cell wells represent expansion clusters, which show the vitality of T cells. In case, BMDMs or T cells do not look as expected, the cells can be incubated for an additional day for settling / expansion in the incubator or the preparation of cell population should be repeated. Use only cell suspensions of optimal conditions for the coculture.

**Figure 3:**
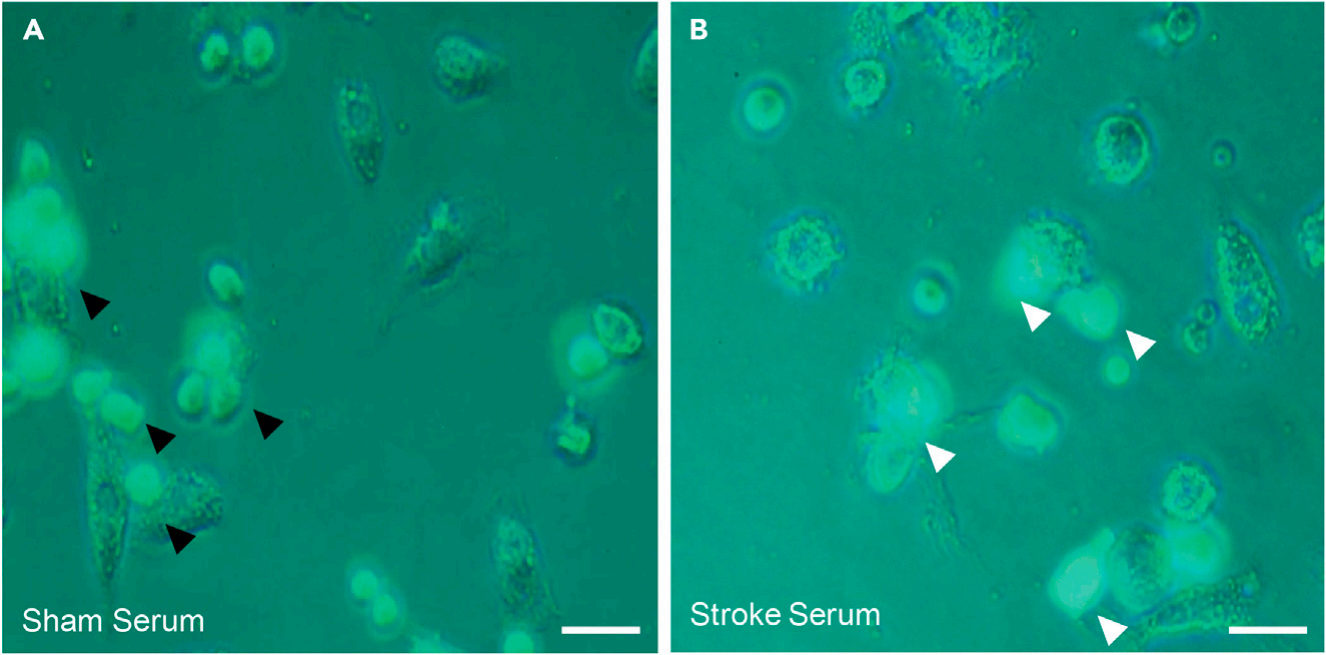
Representative BMDM-T cell coculture images after 90 min stimulation with sham or stroke serum (A) Sham serum-treated BMDMs and roundish shaped, GFP^+^ T cells interact (black arrow heads). (B) Stroke serum-treated BMDMs causing T cell death by cell–cell interaction (white arrow heads). In panel (A) and (B) scale bars represent 50 μm.
  b. **Flow cytometric analysis** of T cell death after coculture stimulation: All cells (per well) are harvested 2–6 h after cell–cell interaction (7.) and stained for surface markers to clearly distinct between macrophages and T cells. Possible markers: T cells (CD3), Macrophages (CD11b, F4/80), Receptor-induced cell death (FasL (CD178)), Live/death marker (Propidium Iodide, Zombie etc.; according antibodies are listed in key resources table). A representative data set can be found in Figure 5.
    ***Optional:*** T cell proliferation, using CFSE, can also be analyzed by flow cytometry and can provide valuable information (e.g. Zhang et al., 2020).
    i. Cells are harvested 2–6 h after the initiation of the cell–cell interaction phase. For the last 10 min of the cell–cell interaction phase, the 96-well plates are placed under the laminar air flow at room temperature.
    ii. Medium in the wells is removed and cells are washed once with 1 mL HBSS and then harvested by carefully utilizing a cell scraper.
    iii. Cells are transferred in 5 mL conic tubes (“FACS tubes”) and volume is brought to 2 mL by adding additional HBSS to cells and held on ice.
    iv. Cells are then washed (300 *g*, 5min). Supernatant is discarded and the cells are resuspended in 100 μL of FACS staining buffer (see key resources table).
    v. Cells are incubated with 1 μL Fc blocker (0.5 μg/mL) for 15 min in the dark at 4°C.
    vi. Antibody cocktail (concentration per antibody: 0.5 μg/mL) is added to the cells (see key resources table).
    vii. Cells are incubated for 30 min in the dark at 4°C.
    viii. After incubation, the cell suspension volume is brought to 2 mL with FACS staining buffer and cells are washed at 300 *g* for 7 min. The supernatant is discarded. Repeat this step once, to improve the cleanness for flow cytometry.
    ix. Bring the cells to a final volume of 200 μL with FACS staining buffer and start acquisition (Representative results in Figure 5).

10. BMDMs: Priming for inflammasome activation.
  a. Dilute 100 ng/mL LPS (see key resources table) in BMDM medium for priming the macrophage’s inflammasome. Incubate for 4 h at 37°C in the incubator (Figures 1B and 1C).
    ***Optional:*** Control the cells visually 2 h after start priming. They should show an expanded nucleus and tend to an amoeboid morphology.
    **Pause point:** During the 4 h of BMDM priming everything for T cells and later-on procedure can be prepared.

11. T cells: Preparation for cell–cell interaction.
  a. Carefully pipette T cells out of U-bottom wells into a 15 mL conic tube prepared with 2 mL pre-warmed T cell medium.
  b. Wash the cells at 300 *g* for 10 min (RT).
  c. Resuspend T cells in 2 mL pre-warmed T cell medium and titrate the concentration appropriate for cell–cell interaction (2 × 10^5^ T cell per well were used in this approach, different T cell concentrations (from 1:1 up to 1:8) are possible.
    ***Optional:*** Keep cells in a conic 15 mL tube and T cell medium briefly before placing them on the stimulated BMDMs.

12. BMDMs: Inflammasome stimulation of BMDMs with murine stroke serum.
  a. Remove carefully the medium from the BMDMs and immediately provide “Inflammasome stimulation medium” (see “before you begin”).
  b. Keep cells in the incubator at 37°C for the stimulation.
    ***Note:*** Ideal stimulation time can vary on the serum. We used serum, collected from mice 4–6 h after severe brain ischemia (Figure 1). This serum strongly activated the inflammasome within 15 min.

13. BMDMs + T cells: Cell–cell interaction.
  a. Carefully remove “Inflammasome stimulation medium” from BMDMs and immediately wash once with 200 μL serum-free RPMI 1640 complete medium.
  b. Add immediately 100 μL serum-free RPMI 1640 complete medium to the washed BMDMs.
  c. Add T cells (2 × 10^5^ cells in 200 μL T cell medium per well, in T cell medium) to the BMDMs. The total volume should be 300 μL (Figure 2).
    ***Optional:*** Centrifuge the 96-well plate at 300 *g* for 1 min to settle the T cells on the BMDMs.
  d. Keep the coculture in the incubator at 37°C until the end of the incubation.

14. Readout recommendations:
  a. **Microscopy-based analysis:** live counting of GFP+ T cells (isolated from B6 actin-eGFP mice, see “preparation of mice”) in distinct fields of view (FOVs; Figure 3) and histology of fixed coculture .

## Expected outcomes

Stroke and other sterile tissue injuries induce a state of systemic immunosuppression. A key characteristic is the induction of lymphopenia, the reduction of lymphocyte counts cells in blood, lymph nodes and spleen (Offner et al., 2006; Liesz et al., 2009; Meisel et al., 2005). We have previously identified that the interaction of innate immune cells (monocytes) with lymphocytes is the critical step in mediating lymphocyte loss after tissue injury (Roth et al., 2021). The *in vitro* model presented here, enables to study the details of this cell–cell interaction.

For bone marrow cell isolation from one mouse (2 femurs and tibiae), expected yield is approximately 6–10 × 10^7^ cells. Healthy cells will be seen as clear, roundish cells in suspension after the cell isolation. Within the first 2–3 days whole spectrum of bone marrow-derived cells, such as erythrocytes and neutrophils, can still be found in the culture. The proliferation and differentiation process of BMDMs, driven by rM-CSF or L929-conditioned medium (L929 murine fibroblasts), provides adherent, mononuclear cells. Erythrocytes and other bone marrow-derived cells disappear. Macrophages will ramify from day 3–4 on and provide a confluent layer of macrophages by day 7 (Figure 2A).

For isolation of splenic T cells using a commercially available enrichment kit (see key resources table), expected yield per murine spleen is approximately 3–5 × 10^7^ cells. Seeding the enriched T cells in anti-CD3e coated 96-well U bottom wells and the addition of anti-CD28, provides active, vital T cells overnight (Figure 2B).

**Figure 4:**
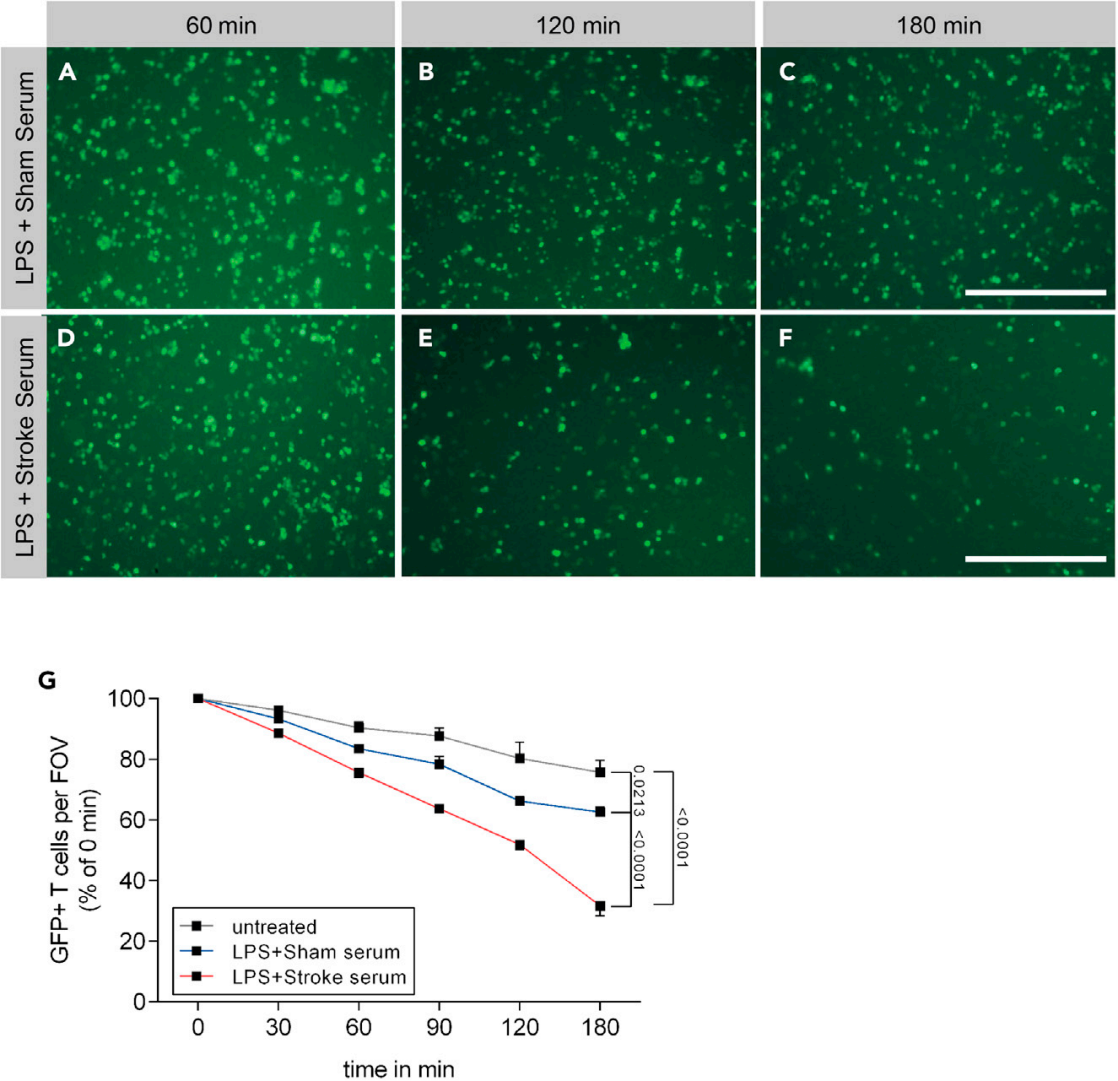
Representative FOV images of BMDM-T cell coculture and GFP^+^ T cell quantification (A–C) GFP^+^ T cells in coculture with BMDMs after of the BMDMs stimulation with serum from sham-operated mice 60, 120 and 180 min after adding T cells. (D–F) GFP^+^ T cells in coculture with BMDMs after stimulation of the BMDMs with serum from stroke-operated mice 60, 120 and 180 min after T cell addition to the activated BMDMs. (G) Normalization of GFP+ T cells to “0:00 timepoint” enables to compare the loss of T cells between different groups over time (Data are represented as mean ± S.E.M.). In panel A to F scale bars represent 500 μm (as seen in C and F).

**Figure 5:**
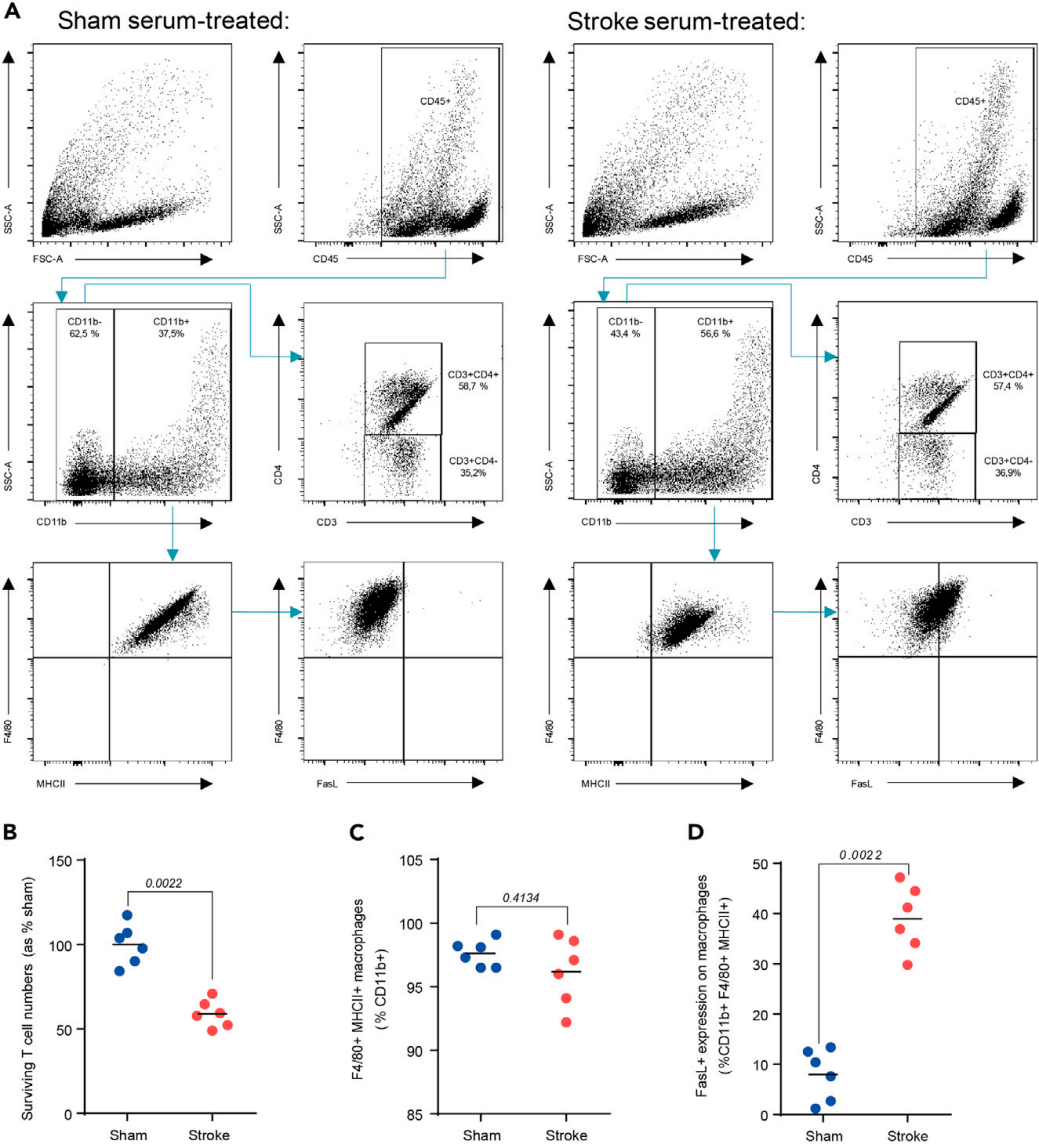
Representative flow cytometric analysis of BMDM – T cell coculture (A) Representative gating strategy for analysis of CD45+CD11b+F4/80+MHCII+ macrophages, FasL abundance and CD45+CD11b-CD3+CD4^+/−^ T cells. (B) T cells analyzed by FACS after cell–cell interaction phase in coculture, initially treated with sham or stroke serum. (C) Proportion of F4/80+MHCII+ macrophages in coculture after serum treatment (sham or stroke). (D) FasL expression on coculture macrophages analyzed by FACS after serum treatment. (n=6 per group; 3 independent experiments).

The activation of BMDMs with an inflammasome stimulus after priming with LPS leads to cleavage of caspase-1, the effector enzyme of the inflammasome (Figures 2C and 2D). Inflammasome activation leads to the release of IL-1β, activating other myeloid cells to express cell death receptor ligands, such as Fas Ligand (FasL; Martin and Wesche, 2002). Activated T cells express the death receptor Fas on their surface (Yamada et al., 2017). In this context, cell–cell interaction is essential for T cell loss (Figure 3). The loss of T cells can be identified by time-lapse microscopy and flow cytometry (Figures 3 and 4). The advantage of the microscopic approach is to identify the starting point of T cell loss. In the above explained setup, a decrease of GFP+ T cells can be observed (treated with murine stroke serum) already 60 min after starting the cell–cell interaction phase (Figure 4). The advantages of flow cytometry are the possibility to acquire absolute cell numbers and the expression of death receptor ligands. In the coculture setup (used ratio Macrophages/T cells: **1:2**), treatment with murine stroke serum shows a decrease of T cells compared to macrophages (CD11b+ / CD11b- percentages; Figures 5A and 5B). The percentage of differentiated macrophages (CD11b+F4/80+MHCII+) did not vary between treatment groups (Figure 5C). The expression of FasL on macrophages was strongly increased after treatment with murine stroke serum compared to sham serum treated coculture (Figure 5D).

In summary, this approach allows the temporal analysis of myeloid cell-induced T cell death after a systemic event of immune activation due to sterile tissue injury.

## Quantification and statistical analysis

### Microscopy based analysis

During the cell–cell interaction phase (in this approach 180 min) we acquired microphotographs of determined fields of views from every well of the triplicates. Single channel (GFP) acquisition was sufficient to analyze T cell numbers. The next step was thresholding (setting the range for true-positive fluorescence) and binarizing the photographs in ImageJ. This enables to use the “analyze particles” function of the software. Based on magnification and resolution of the images, particle size and sphericity values are set. Finally, quantification of the binarized images lead to a single cell number value per FOV, which then was used for further statistical analysis.

### Flow cytometric analysis

For the last 10 min of the cell–cell interaction phase, the 96-well plates are placed under the laminar air flow at room temperature. Cells were carefully harvested by cell scraping and collected in HBSS. Cells are then stained with the according flow cytometry antibodies (see key resources table). After the wash step cells are brought to the same volume and acquired with a multicolor flow cytometer with volumetric measurement. We collected 10,000 CD45+ cells per sample which enabled us to calculate back to the total cell amount per well. To determine the two cell types from the well (macrophages and T cells) we gated for CD45+CD11b+F4/80+MHCII+ macrophages and CD45+CD11b-CD3+CD4^+/−^ T cells (Figures 5A–5C). The ability of macrophages to induce T cell apoptosis was quantified by the amount of FasL-expressing (CD178+) macrophages (Figure 5D).

## Limitations

The coculture model mimics the complex interactions between innate and adaptive immune cells after a sterile tissue injury. Although the model provides high reproducibility, it brings along a number of limitations.

The number of cells and cell types is limited, unlike in the *in vivo* models. As the coculture facilitates the pool of splenic CD3+ T cells and to rM-CSF differentiated bone marrow-derived macrophages, the variety of cells is of course restricted in comparison to a diverse systemic pool of immune cells which egress and migrate during local and systemic inflammation. While this model enables to observe the interaction between two different cell populations, additional remote interactions with other cell populations and the complex immunological environment via cytokines and chemokines cannot be modeled. Also immunological effects on remote organs or the important aspect of secondary infections cannot be investigated with this model.

This coculture is optimized for monocytic inflammasome activation which subsequently leads to T cell death – the predominant way of T cell death after sterile tissue injury (Roth et al., 2021). However, the role of specific (murine) T cell-expressed inflammasome sensors, such as CARD8 (Linder et al., 2020), need a different experimental design.

The mentioned approaches to quantify T cell death in coculture – microscopy and flow cytometry – also include limitations: Time lapse microscopy allows a fast and repetitive readout. However, further cellular information can only be acquired by fixation and additional staining of potentially interesting target proteins. Flow cytometry can provide a multitude of additional information dependent on the used antibodies. However, only one time point per sample can be analyzed, due to the process of collection and staining of cells. Moreover, a lengthy or faulty procedure of staining living cells can affect the survival of cells and by that, affect the experimental outcome.

## Troubleshooting

### Problem 1

Only a little number of cells can be found after culturing bone marrow-derived cells for BMDM differentiation (steps 1–4 in “generation of murine bone marrow-derived macrophages (BMDMs)”).

### Potential solution

Make sure a pellet can be seen after every washing step during the bone marrow cell collection. After titrating cells for seeding, check cell densities on the dishes. Careful expose and extract femur and tibia and do not damage the bones. Detailed information can also be found in Toda et al., (2020).

### Problem 2

Inflammasome activation is not sufficient with donor serum (steps 10–14 in “coculture procedure”).

### Potential solution

Make sure the serum donor mice had a sufficiently severe tissue injury. Only minor lesions such as small, cortical brain injuries do not lead to sufficient release of inflammasome-activating molecules in the serum. Concentrations of potential inflammasome activators in the serum, like dsDNA, can be acquired beforehand. For excluding problems with the BMDMs, the use of positive control for inflammasome activation (e.g., 10 μM Nigericin) can be helpful.

### Problem 3

Cell culture shows bacterial contamination (“moving particles” under the microscope) (steps 5 and 6 in “harvest and seeding of BMDMs in 96-well plates” and steps 7–9 in “T cell isolation from murine spleen”).

### Potential solution

Be aware that all tools are sterilized and stored in ethanol. Soak the mice skin with ethanol when collecting bones and spleen for cell culture to avoid contaminations. All operations have to be conducted under a laminar air flow hood. Check the status of the cells every day. Once potentially contamination of bacteria, fungi, or recombinant protein is found, discard the cells and appropriately autoclave all cell culture dishes and media.

### Problem 4

Only a small amount of BMDMs can be detected after the coculture approach (steps 10–14 in “Coculture procedure”).

### Potential solution

Two aspects need to be taken under consideration: 1. Activation of the cells (LPS priming and later stimulation) lead to morphological changes of the macrophages and potential loss of adherence. 2. (Over) activation of the inflammasome can lead to pyroptotic cell death of the macrophages. When establishing a new stimulus, perform a titration experiment to determine the least necessary stimulus concentration in order to avoid cell toxicity.

### Problem 5

Cells and/or whole well content clots after adding the murine serum (see “murine stroke serum collection and storage” in “materials and equipment”.)

### Potential solution

The initial preparation of the serum was unsuccessful. Check during preparation, if the blood is clotted after the 30 min incubation step at room temperature. If blood is not fully clotted add another 10 min of incubation at room temperature.

## Resource availability

### Lead contact

Further information and requests for resources and reagents should be directed to and will be fulfilled by the lead contact, Stefan Roth (stefan.roth@med.uni-muenchen.de).

### Materials availability

No materials were newly generated for this protocol. All materials mentioned above are commercially available.

## Data Availability

Datasets generated with this protocol can be found in Roth et al., (2021). Raw data used in this article is found in “Table 1”.

**Table 1:** Example of GFP+ T cell count from coculture

Time min	Raw data cell numbers per FOV								
	UT 1	UT 2	UT 3	Sham serum (25%) 1	Sham serum (25%) 2	Sham serum (25%) 3	Stroke serum (25%) 1	Stroke serum (25%) 2	Stroke serum (25%) 3
0:00	401	351	300	398	376	401	423	399	410
30:00	383	336	292	379	351	383	374	351	367
60:00	360	329	263	324	323	354	318	314	300
90:00	348	325	251	293	303	332	271	260	254
120:00	330	310	211	256	245	282	212	220	207
180:00	300	291	200	240	234	263	156	171	104

Data was analyzed using ImageJ software. Microphotographs (Figure 4) from the coculture kinetics were thresholded for GFP+ cells. Thresholded cells were then quantified using “analyze particles” task. All acquired numbers of GFP+ cells were normalized to the according “0:00” cell numbers and plotted in a graph (Figure 4). Abbreviations: UT = untreated Example of GFP+ T cell count from coculture Data was analyzed using ImageJ software. Microphotographs (Figure 4) from the coculture kinetics were thresholded for GFP+ cells. Thresholded cells were then quantified using “analyze particles” task. All acquired numbers of GFP+ cells were normalized to the according “0:00” cell numbers and plotted in a graph (Figure 4). Abbreviations: UT = untreated
